# Effects of Pyriproxyfen Exposure on Reproduction and Gene Expressions in Silkworm, *Bombyx mori*

**DOI:** 10.3390/insects11080467

**Published:** 2020-07-24

**Authors:** He-Ying Qian, Xiao Zhang, Guo-Dong Zhao, Hui-Min Guo, Gang Li, An-Ying Xu

**Affiliations:** 1 School of Biotechnology, Jiangsu University of Science and Technology, Jiangsu 212018, China; zhangxiao10256@163.com (X.Z.); sdgdzhao@just.edu.cn (G.-D.Z.); 15735531904@163.com (H.-M.G.); gangsri@just.edu.cn (G.L.); 2 Sericultural Research Institute, Chinese Academy of Agricultural Sciences, Jiangsu 212018, China

**Keywords:** *Bombyx mori*, gene expression, hormone regulation, ovary development, reproduction

## Abstract

The silkworm, *Bombyx mori* Linnaeus, is an important economic insect and a representative model organism of Lepidoptera, which has been widely used in the study of reproduction and development. The development of the silkworm’s reproductive gland is easily affected by many external factors, such as chemical insecticides. After the silkworm larvae were treated with different concentrations of pyriproxyfen, the results showed that the number of eggs and hatching rate of eggs in the silkworm can be reduced by pyriproxyfen, and the concentration effects were displayed. Pyriproxyfen exposure could affect the normal development of the ovary tissue by reducing the number of oocytes and oogonia in the ovaries of silkworm fed with pyriproxyfen. We employed qRT-PCR, to detect the expressions of genes related to ovary development (*Vg*, *Ovo, Otu*, *Sxl-S* and *Sxl-L*) and hormone regulation (*EcR* and *JHBP2*) in silkworm. Our study showed that the transcription levels of *Vg*, *Ovo, Otu*, *Sxl-S* and *Sxl-L* in the treatment group were lower than those in the control group (6.08%, 61.99%, 83.51%, 99.31% and 71.95%, respectively). The transcription level of *ECR* was 70.22% for the control group, while that of *JHBP2* was upregulated by 3.92-fold. Changes of transcription levels of these genes caused by pyriproxyfen exposure ultimately affect the absorption of nutrients, energy metabolism, ovary development and egg formation of the silkworm, thus leading to reproductive disorders of the silkworm. In general, our study revealed the response of silkworm reproduction to pyriproxyfen exposure and provided a certain reference value for the metabolism of the silkworm to pyriproxyfen.

## 1. Introduction

Pyriproxyfen is an insect growth regulator (IGR) that disrupts insect development at specific stages and is a juvenile hormone mimic. It is a mature biological pesticide with juvenile hormone activity in insect growth regulators that has the characteristics of strong light stability, long-term field efficacy and low toxicity to mammals. Pyriproxyfen can affect the reproduction of insects by making insects lack the stimulation factors needed for spawning or inhibiting the development of egg and embryo directly or indirectly [1,2]. In integrated pest management (IPM) programs, chemical control is still the most commonly used method, especially when there is a large occurrence of pests and other control methods cannot work immediately, chemical control can quickly reduce the population density of pests without regional and seasonal restrictions, and the effect is remarkable. Pyriproxyfen is considered to be one of effective insecticide to pest control in crop systems [3]. The study on the application of pyriproxyfen in the control of agricultural pests showed that pyriproxyfen had no significant effect on the growth of larvae and the fecundity of *Chilacarus nigritus* (Fabricius) adults, but the eggs exposed to pyriproxyfen could not survive [4]. Chen and Liu also reported that pyriproxyfen can cause 95.3–100% of *Chrysoperla rufilabris* (Burmeister) eggs to be difficult to develop into adults [5]. It was shown that pyriproxyfen had a significant killing effect on the eggs of *Bemisia tabaci* [6], and 100 PPM of pyriproxyfen had a lethal effect on 94.5% of *Bemisia tabaci* eggs [7].

The silkworm is a kind of oligotrophic silk spinning insect with high economic value, and it feeds on mulberry leaves, which are the most suitable natural food for silkworms. In recent years, pesticide poisoning of silkworms has occurred from time to time. With the continuous development and application of new pesticides, the threat of silkworm poisoning is growing, and the diagnosis of poisoning symptoms and preventive measures are becoming more and more difficult. In the spring of 2019, more than half of silkworms could not spin or died due to the application of insecticides, including pyriproxyfen in Luliang County, Yunnan Province. It has been reported that pyriproxyfen not only has thin-layer-transfer ability in the leaves, but also has a good upward conduction absorption effect [8]. The toxic degradation of pyriproxyfen on mulberry leaves is very slow. A small amount of pyriproxyfen treatment can cause the silkworm to eat mulberry leaves more slowly, prolong the larval age and increase the weight during molting stage. Even if the larvae can molt, the function of cocooning is destroyed, and the cocoon cannot be formed [9,10]. However, up until now, there has been no report about the harm of pyriproxyfen to silkworm eggs.

The silkworm is a kind of complete-metamorphosis insect, which has four important stages in its life cycle: egg, larva, pupa and adult. The egg plays an important role in the process of embryo development. The reproductive function of silkworms is not only affected by the external environment and exogenous chemicals, but also by the expression level of relevant functional genes. Vitellogenin (Vg) is a high-volume protein synthesized in female insects. It is synthesized by fat body and transported to the ovary through circulation of hemolymph, providing nutrition for the formation and development of the embryo [11]. Lin et al. found that a large amount of yolk protein in the eggs was reduced by injecting the interference fragment of the Vitellogenin receptor (VgR), which resulted in the inadequate nutrition of the embryos in the eggs and abnormal development [12]. OVO transcription factors are a group of evolutionarily conserved protein families encoded by the *Ovo* gene, which plays an important role in the growth and development of organisms. In germ cells, OVO can regulate the expression of ovarian tumor gene (*Otu*) by binding to the promoter of *Otu* directly and regulate the development of ovary and germ cells [13,14]. *Otu* plays various functions in the formation of female germ cells, such as regulating the formation of fusion body, which is related to the formation of actin bundles [15,16]. Sex determination is the result of evolutionary selection of species and the basic characteristics of insect life activities, which can affect all aspects of individual development, such as embryogenesis, sex differentiation, reproduction and other physiological processes [17]. Sxl is the regulatory center of sex determination and the hinge of germ cell differentiation [18].

The reproduction of female insects is controlled by different kinds of hormones, including 20-hydroxyecdysone (20E), juvenile hormone (JH) and neuropeptide. The typical steroid hormone 20E is involved in regulating the physiological processes of molting, metamorphosis and reproduction [19]. The heterodimer complex formed by the combination of ecdysone receptor (ECR) and ultra-valve protein (USP) is the target of 20E [20]. Ecdysteroid receptor (EcR), a member of the steroid receptor superfamily, plays an important role in the regulation of silkworm reproduction and other life activities [21]. It has been shown that the expression of *Vg* in the silkworm is mainly regulated by 20E [22]. Bownes et al. found that the synthesis of 20E is necessary for the activation and maintenance of vitellin gene transcription [23]. JH is an intrinsic endogenous hormone of insects which can regulate the development, metamorphosis and reproduction of insects as an insect gonadotropin [24]. Juvenile hormone binding protein (JHBP) is a carrier for transporting JH and function in vivo, which can be transported to target tissue such as ovary with circulation of hemolymph in the form of complex with JH [25]. Previous studies have shown that USP is a potential JH receptor, and the expression characteristics of USP are related to the changes of JH [26].

Although the trace of pyriproxyfen will not cause acute poisoning to the silkworm, it can affect the growth and development of the silkworm and its cocooning function. At present, there is no report about the effect of pyriproxyfen on oviposition and hormone regulation of silkworms. In this study, we evaluated the effect of trace amounts of pyriproxyfen exposure on the expression level of ovary-development-related genes and the effect on the mechanism of 20E and JH coordinated regulation, and we elucidated the response of silkworm ovary to pyriproxyfen exposure. Our results can provide data and theoretical support for the harm of pyriproxyfen exposure to the reproduction of the silkworm, and they have certain reference value for the prevention of pesticide pollution in sericulture.

## 2. Materials and Methods

### 2.1. Insect Strains

The silkworm strain Jingsong was preserved in Sericultural Research Institute, Chinese Academy of Agricultural Sciences. Hatching of silkworm eggs: The room temperature from the first to the fourth day was 24 °C, the difference between dry and wet was 2–2.5 °C, the room temperature from the fifth to the tenth day was 27 °C and the difference between dry and wet was 1.5–2 °C. Larva feeding: The larvae were reared with mulberry leaves in the condition of 28 ± 1 °C and 85% relative humidity at the age from 1st instar to 3rd instar, and 25 ± 1 °C and 70% relative humidity at the age from 4th instar to 5th instar.

### 2.2. Chemicals

Pyriproxyfen was purchased from Sigma-Aldrich Company (Shanghai, China). IUPAC chemical name: 4-phenoxyphenyl (RS)-2-(2-pyridyloxy) propyl ether, 95% purity. In order to ensure the survival of the silkworm, the dose of pyriproxyfen (0.001 mg/L) that we used was far lower than the recommended concentration in the field [9]. The stock solution was diluted with ddH_2_O to a final concentration of 100 mg/L, to prepare a working solution. The mulberry leaves were treated by the immersion method: The mulberry leaves were soaked in the working solution for 1 min and then dried naturally.

TRIzol, chloroform, isopropyl alcohol, SYBR premix, ROX and other chemicals were purchased from Takara (Dalian, China). The primers were synthesized by Shanghai Sangon Biological Technology and Services Co., Ltd. (Shanghai, China).

### 2.3. Sample Preparation

The 1st to 4th instar larvae were fed with fresh mulberry leaves. At the 5th instar, the larvae were fed with fresh mulberry leaves from the 1st to 3rd day, and on the fourth day, the larvae were divided into three control groups and three treatment groups. There were 120 larvae for each group, of which 60 were used for statistical data and 60 for anatomical materials. In the control group, the larvae were fed with mulberry leaves treated with H_2_O from the 4th day 5th instar until mounting, and in the treatment group, the larvae were fed with mulberry leaves treated with 0.001 mg/L pyriproxyfen until mounting. At 0, 24, 48, 72 and 96 h after treatment, the larvae were dissected, and the ovary was isolated and stored at −80 °C. After eclosion, the healthy male and female moths were selected and put together for mating. Then, 6 h later, the male and female moths were separated, and the female moths were put in the egg batch for laying eggs. Twelve hours later, the female moths were killed, and the eggs were counted.

### 2.4. Histopathological Examination

The ovary was randomly collected and fixed in 4% glutaraldehyde, and then embedded in paraffin. The slices (5 μm) were cut from paraffin blocks and then fixed on the flakes. The sections were stained with hematoxylin–eosin and observed under optical microscopy. All histological examinations were performed according to the methods in previous research [27].

### 2.5. Isolation of Total RNA

TRIzol reagent (Takara, Dalian, China) was used to extract total RNA from the ovary. The isolated RNA was treated with DNase (Takara, Dalian, China), to remove potential genomic DNA contamination. First-strand cDNA was synthesized with M-MLV reverse-transcriptase (Takara, Dalian, China) and oligo (dT) primer (Sangon, Shanghai, China). The quality of RNA was assessed by formaldehyde agarose gel electrophoresis and was quantified spectrophotometrically. After pyriproxyfen exposure, the concentrations of RNA in control group and treatment group were more than 1000 ng/μL. The OD_260/280_ in the control group and treatment group was 1.90–2.00 after pyriproxyfen exposure.

### 2.6. Quantitative RT-PCR (qRT-PCR)

In this study, SYBR Prime Script^TM^ RT-PCR kit (TaKaRa, Dalian, China) was used for the determination on ABI Prism 7300 Fluorescence Quantitative PCR Instrument (Applied Biosystems, Foster City, CA, USA). The specific experimental steps of primers used for real-time qRT-PCR (Table 1) were designed by using Primer (6.0) software, in accordance with the instructions, and the reaction system was 20 μL. Amplification conditions were as follows: denaturation at 95 °C for 1 min, followed by 40 cycles of 95 °C for 15 s, 55 °C for 10 s and 72 °C for 10 s. Data were expressed as the mean ± SE (standard error) of three independent experiments. The transcriptional levels of genes were calculated according to the 2^−ΔΔCt^ method [28].

### 2.7. Statistical Analysis

The results are shown as the mean ± standard error (SE) of three independent measurements. To determine the effect of pyriproxyfen exposure on the reproduction of silkworms and expression-level changes of genes, one-way ANOVA was performed by different concentrations and exposure times, for comparisons between groups. Asterisks denote significant differences as compared with the control group, as indicated by * *p* ≤ 0.05 and ** *p* ≤ 0.01.

## 3. Results

### 3.1. The Effects of Pyriproxyfen on the Oviposition of Silkworm at Larvae Stage

**Figure 1:**
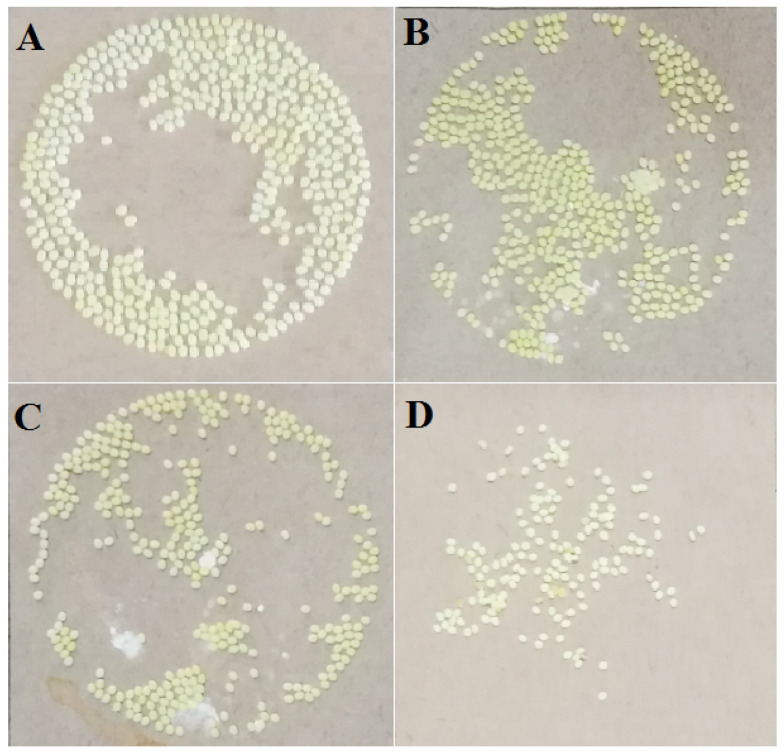
The oviposition of silkworms from different treatment groups: (**a**) control group, (**b**) 0.001 mg/L of pyriproxyfen treatment group, (**c**) 1 mg/L of pyriproxyfen treatment group and (**d**) 100 mg/L of pyriproxyfen treatment group.

The oviposition of silkworm can be affected by pyriproxyfen exposure. The results showed that 459 eggs were laid by a silkworm moth in the control group (Figure 1a), and the average number of eggs decreased to 408 eggs/moth (Figure 1b); accordingly, the average hatching rate decreased from 98.47% in the control group to 92.44% in the 0.001 μg/L group. After the treatment with 1 μg/L pyriproxyfen, the average number of eggs laid by a silkworm moth decreased to 370/moth (Figure 1c), and the average hatching rate decreased to 89.43%. After the treatment of 100 μg/L pyriproxyfen, the number of eggs laid by the silkworm moth decreased sharply, with an average of 142 eggs/moth (Figure 1d), and the average hatching rate also decreased sharply to 5.20%. The results showed that the effect of pyriproxyfen exposure on laying eggs was a concentration effect, that is, the greater the concentration of pyriproxyfen, the more significant the effect (*F*_3,56_ = 4.3; *p* = 0.008). In addition, the rate of fertilized eggs was also affected by exposure of pyriproxyfen, and the concentration effect can be seen from the data. Furthermore, compared to the control group, the size of an individual egg from the pyriproxyfen-treated group was also much smaller. However, the amount of eggs laid and the hatchability of eggs decreased significantly after the exposure of 100 μg/L of pyriproxyfen, which indicated that pyriproxyfen could seriously damage the normal reproductive function of the silkworm (Table 2).

### 3.2. The Pathological Evaluation of the Ovary after Pyriproxyfen Exposure

**Figure 2:**
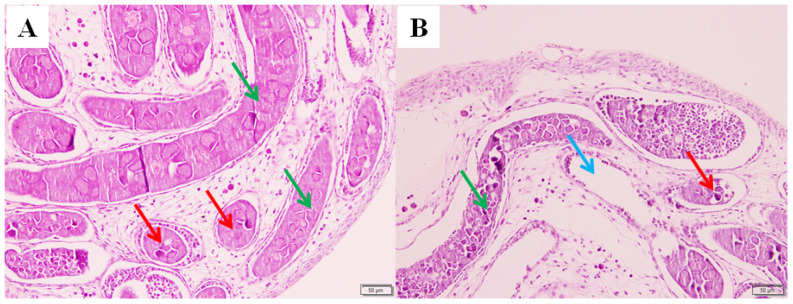
Histological images of ovary tissue in fifth-instar *B. mori* larvae at 96 h after pyriproxyfen exposure. (**a**) Control group (100×) and (**b**) 0.001 mg/L of pyriproxyfen treatment group (100×). The green arrow indicates the oocyte, the red arrow indicates the oogonia and the blue arrow indicates the formation of vacuoles.

Compared with the control group, it was found that the number of oocytes and oogonia in the ovaries of the silkworm fed with pyriproxyfen decreased, and some vacuoles appeared in the ovaries of the treatment group. The results showed that pyriproxyfen exposure could affect the normal development of the ovary tissue of the silkworm, which might lead to the abnormal reproductive function of the silkworm (Figure 2).

### 3.3. The Effect of Pyriproxyfen Exposure on the Expression Level of Vg in the Ovary

**Figure 3:**
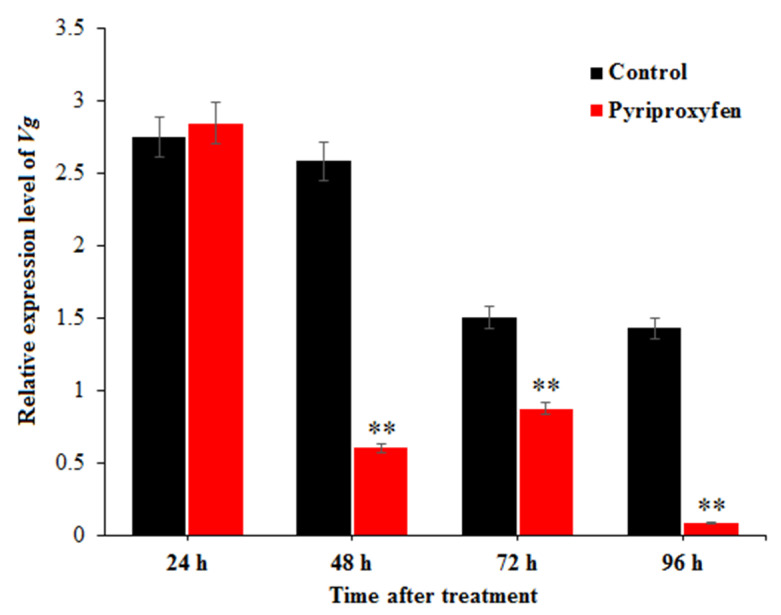
Expression-level changes of *Vg* in the ovary of silkworm at different times after pyriproxyfen exposure. The black histogram represents the control group, and the red histogram represents the treatment group. The *x*-axis represents the time after pyriproxyfen exposure, and the *y*-axis represents the relative expression level of *Vg*. The results are shown as the mean ± SE. Asterisks denote significant differences between treatments and controls, as indicated by * *p* ≤ 0.05 and ** *p* ≤ 0.01.

The relative expression levels of *Vg* in the ovaries of silkworms were measured by qRT-PCR, and the results are shown in Figure 3. Compared to control group, the expression level of the *Vg* gene in the treatment group was significantly decreased at 48 h (*F*_1,16_ = 10.5; *p* = 0.005), 72 h (*F*_1,16_ = 9.7; *p* = 0.007) and 96 h (*F*_1,16_ = 12.3; *p* = 0.003) after treatment (23.33%, 58.24% and 6.08%, respectively). At 96 h, the relative expression of *Vg* in the treatment group was the lowest. It was found that pyriproxyfen exposure can reduce the expression of *Vg* in the ovary of a silkworm and cause the decrease of yolk protein synthesis, thus leading to the decrease of nutrients for embryonic development.

### 3.4. Transcriptional Analysis of Genes Related to Ovarian Development after Pyriproxyfen Exposure

**Figure 4:**
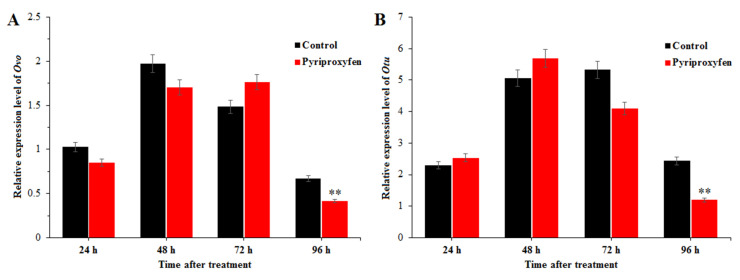
The effect of pyriproxyfen exposure on the expression of genes related to ovarian development: (**a**) *Ovo* and (**b**) *Otu*. The black histogram represents the control group, and the red histogram represents the treatment group. The *x*-axis represents the time after pyriproxyfen exposure, and the *y*-axis represents the relative expression level of genes. The results are shown as the mean ± SE. Asterisks denote significant differences between treatments and controls, as indicated by * *p* ≤ 0.05 and ** *p* ≤ 0.01.

In order to evaluate the effect of pyriproxyfen exposure on the ovarian development of the silkworm, the transcription levels of *Ovo* and *Otu* were detected by using real-time qRT-PCR. The results indicated that the expression levels of these two genes showed the same change trends after pyriproxyfen exposure (Figure 4), that is, the transcription levels change little at 24, 48 and 72 h, but can be significantly downregulated at 96 h after treatment (*F*_1,16_ = 8.8; *p* = 0.009; *F*_1,16_ = 9.1; *p* = 0.008).

### 3.5. The Effect of Pyriproxyfen Exposure on the Expression Levels of Genes Related to Sex Differentiation in the Ovary

**Figure 5:**
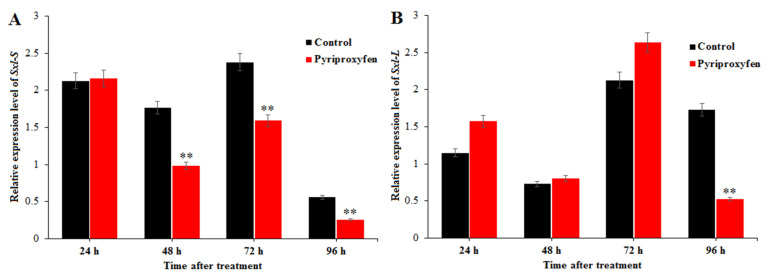
The effect of pyriproxyfen exposure on the expression levels of genes related to sex differentiation: (**a**) *Sxl-S* and (**b**) *Sxl-L*. The black histogram represents the control group, and the red histogram represents the treatment group. The *x*-axis represents the time after pyriproxyfen exposure, and the *y*-axis represents the relative expression level of genes. The results are shown as the mean ± SE. Asterisks denote significant differences between treatments and controls, as indicated by * *p* ≤ 0.05 and ** *p* ≤ 0.01.

Relative expression level of *Sxl-S* in the treatment group was lower than that in the control group at 48 h (*F*_1,16_ = 9.2; *p* = 0.008), 72 h (*F*_1,16_ = 8.9; *p* = 0.009) and 96 h (*F*_1,16_ = 8.7; *p* = 0.009) after pyriproxyfen exposure (55.48%, 66.90% and 45.61% respectively). The relative expression level of *Sxl-L* showed little changes at 24, 48 and 72 h after pyriproxyfen exposure, and it was significantly downregulated at 96 h with the ratio of 30.37% (*F*_1,16_ = 11.2; *p* = 0.004; Figure 5). The results indicated that pyriproxyfen can affect the development of ovary, and its mechanism is mainly through affecting the transcriptional expression of these related genes.

### 3.6. Changes in the Expression Levels of Genes Related to 20E and JH Regulation in Pyriproxyfen Treated Silkworms

**Figure 6:**
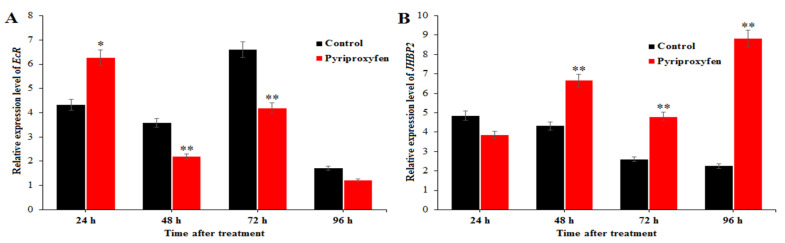
The effect of pyriproxyfen exposure on the expression of genes related to ovarian development: (**a**) *EcR* and (**b**) *JHBP2*. The black histogram represents the control group, and the red histogram represents the treatment group. The *x*-axis represents the time after pyriproxyfen exposure, and the *y*-axis represents the relative expression level of genes. The results are shown as the mean ± SE. Asterisks denote significant differences between treatments and controls, as indicated by * *p* ≤ 0.05 and ** *p* ≤ 0.01.

Relative expression level of *EcR* in the ovary was detected from 24 to 96 h after pyriproxyfen exposure, and the results are shown in Figure 6. Relative expression level of *EcR* in the treatment group at 48 h (*F*_1,16_ = 5.9; *p* = 0.027), 72 h (*F*_1,16_ = 9.5; *p* = 0.007) and 96 h (*F*_1,16_ = 9.9; *p* = 0.006) after pyriproxyfen exposure was lower than that in the control group with ratio of 61.13%, 63.51% and 70.22%, respectively.

In order to detect the effect of pyriproxyfen exposure on JHBP, the *JHBP2* gene was chosen and detected at 24, 48, 72 and 96 h after pyriproxyfen exposure. At 24 h, the relative expression level of *JHBP2* in the treatment group was lower than that in the control group with ratio of 79.28%. However, the relative expression level of *JHBP2* was significantly elevated at 48 h (*F*_1,16_ = 10.4; *p* = 0.005), 72 h (*F*_1,16_ = 10.1; *p* = 0.006) and 96 h (*F*_1,16_ = 12.5; *p* = 0.003) after pyriproxyfen exposure, with folds of 1.54, 1.84 and 3.92, respectively.

## 4. Discussion

Due to the close distance between mulberry garden and farmland, mulberry leaves are easily poisoned by pesticides, leading to the limitation of sericulture development [29,30]. The ovarian development of the silkworm is easily affected by temperature, starvation, pH, high pressure infiltration and chemical pesticides. Pyriproxyfen is a new type of insect growth regulator which belongs to phenyl ether insecticide [31]. It causes the death of insects by inhibiting embryo development, interfering with molting and blocking metamorphosis. It has been reported that pyriproxyfen can make females lay less eggs and has strong egg-killing activity. Begum et al. found that endosulfan and heptachlor can cause abnormal egg laying and hatching of eggs [32]. The exposure of carbaryl, dichlorvos and cypermethrin to silkworms can also cause reproductive development disorder in silkworms [33,34]. However, the toxicity of pyriproxyfen to the reproductive system of silkworms has not been reported. In the present study, the results indicated that the number of eggs and the hatching rate of eggs decreased after pyriproxyfen exposure, and the concentration effect can be seen from the results.

The development of ovary and egg production of silkworms is regulated by many genes. The embryonic development of silkworms is a process with strong energy demand, in which the yolk protein stored in eggs is consumed to provide energy and nutrients for the embryonic development. Vitellogenin (Vg) is a high-volume protein synthesized by females. After synthesis, it is ingested by the developing oocyte through the endocytosis mediated by vitellogenin receptor (VgR), providing nutrients and energy reserve for future embryo development [35]. After the expression of *Vg* was interfered by RNAi in female pupae, the egg formation and embryo development were both affected, indicating that *Vg* played an important role in this process [36]. In this study, the results showed that the expression levels of *Vg* were downregulated at 48, 72 and 96 h after treatment. It was speculated that the vitellin synthesis was reduced because of the downregulation of *Vg*, leading to the decrease of nutrients and energy in the development of ovarian embryos, and this maybe one of the reasons why the number of eggs and the hatching rate decreased after pyriproxyfen exposure.

*Ovo* and *Otu* are closely related to the normal development of ovarian tissue in insects. OVO protein is a transcription factor, a member of zinc finger protein family, which is very conservative in evolution. Xue used RNAi to interfere the expression of *Ovo* gene, resulting in a significant reduction in the number of eggs laid by about 15%, and accompanied by a decrease in hatchability [37]. At present, it is known that *Otu* plays an important role in the ovarian development of *Drosophila*, and the mutation of *Otu* gene will disturb the normal process of egg formation. It has been reported that OVO protein can activate *Otu* during the ovarian development of *Drosophila* [13]. It binds to a large number of sites near the Otu promoter, and it regulates *Otu* directly [38]. Our results showed that the relative expression level of *Ovo* and *Otu* in the treatment group was the lowest at 96 h, only 61.99% and 49.17% of that in the control group, respectively. *Sxl-S* and *Sxl-L* are self-cutting regulatory genes. It has been shown that the variable cutting of the female-specific *Sxl* gene also plays an important role in the development of the ovary [39]. In the present study, qRT-PCR was used to detect the relative expression of *Sxl-S* and *Sxl-L* in the ovary, and the results indicated that the relative expression levels of *Sxl-S* and *Sxl-L* in the treatment group were lower than those in the control group. It was speculated that the self-regulation of the silkworm caused by pyriproxyfen is abnormal and eventually leads to the abnormal development of ovary.

The oogenesis of insects is the process of egg maturation, yolk formation and accumulation. The development of ovary and oogenesis is regulated by endogenous hormones, including 20-hydroxyecdysone (20E) and juvenile hormone (JH). Studies have shown that the ovarian development is generally induced by 20E shortly after larval molting [40]. Ecdysone receptor (EcR) is a transcription factor which is closely involved in the regulation of vitellogenesis. ECR and ultraspiracle (USP) are all members of nuclear receptor superfamily. When ECR and USP combine to form heterodimer, 20E can be combined to ECR. It can regulate the expression of downstream genes, and then regulate the growth, development and reproduction of insects. In order to understand the effect of pyriproxyfen on 20E in a silkworm’s ovary, we used qRT-PCR to detect the relative expression level of *ECR*. The results showed that the relative expression level of *ECR* in the treatment group was lower than that in the control group, from 48 to 96 h after treatment (61.13%, 63.51% and 70.22%, respectively). That is to say, pyriproxyfen inhibited the expression of *ECR* and the formation of ECR/USP dimer, thus affecting the development of ovary. Juvenile hormone binding protein (JHBP) is the carrier of JH transport and function in vivo [25,41]. In silkworms, JH is synthesized and secreted into hemolymph through the corpus allatum, which can be combined with JHBP and transported to different target organs, such as the ovary, thus regulating the growth and development of the silkworm [42]. The results of qRT-PCR showed that the relative expression level of *JHBP2* in the treatment group was higher than that in the control group at 48, 72 and 96 h after pyriproxyfen exposure. It has been found that USP protein may be one of the potential receptors of JH. After USP and ECR form dimer, 20E can promote the development of the ovary. Therefore, we speculated that, even though the relative expression of *JHBP2* was increased after pyriproxyfen exposure, pyriproxyfen could still reduce the number of eggs and hatching rate due to the inhibition of ECR/USP dimer.

## 5. Conclusions

This study indicated that the number of eggs and the hatching rate of silkworm can be reduced after pyriproxyfen exposure. Pyriproxyfen exposure could affect the normal development of the ovary tissue by reducing the number of oocytes and oogonia in the ovaries of silkworms fed with pyriproxyfen. We used qRT-PCR to detect the genes related to ovary development (*Vg*, *Ovo, Otu*, *Sxl-S* and *Sxl-L*) and hormone regulation (*EcR* and *JHBP2*) in silkworms. The results showed that the transcription levels of *Vg*, *Ovo, Otu*, *Sxl-S* and *Sxl-L*) in the treatment group were lower than those in the control group at 96 h after treatment. The transcription level changes of these genes caused by pyriproxyfen ultimately affected the absorption of nutrients, energy metabolism, ovarian development and egg formation in the silkworm’s ovary, thus leading to reproductive disorders of the silkworm. The transcription level of *ECR* was downregulated, and that of *JHBP2* was upregulated after pyriproxyfen exposure, further inhibiting the development of the ovaries of silkworms fed with pyriproxyfen. In general, our study revealed the response of silkworm reproduction to pyriproxyfen exposure and provided a certain reference value for the study of the metabolism mechanism of the silkworm to pyriproxyfen.

## Figures and Tables

**Table 1:** Primer sequence for qRT-PCR.

Gene Name	Primer Sequence (5’–3’)
*GAPDH*	F: TGTTGAGGGCTTGATGAC
	R: ACCTTACCCACAGCTTTG
*Vg*	F: CTGCAACGCAAGGAAACCAA
	R: TGGCCGTACTTGAAGTGCAT
*Ovo*	F: GCAGCTGCTTTAGGACTACCAG
	R: CGTTAGCTTCAGTCGCCAAA
*Otu*	F: AACCACAACGCTGACCAGAA
	R: GTGGCCCTTGTTCTGATGGT
*Sxl-S*	F: CGCGTTACCTATTTAACATTTCGTG
	R: CCTCGGTACTGCTGTTGGAT
*Sxl-L*	F: CGGGATACTTGTTTGGTGGC
	R: AGACATGCTGCCCCAGTATC
*EcR*	F: TGATGGAGCAGAACAGGCAG
	R: CCTCTTCATCCGACTGCGTT
*JHBP2*	F: CAATGCCTTAGCAGTGCGAC
	R: TGAAGCGTATCACGACTCCC

**Table 2:** Effects of pyriproxyfen exposure on the reproduction of silkworms.

Concentration of Solution/(μg/L)	Average Number of Eggs/(Grain)	Average Number of Hatching Eggs/(Grain)	Hatching Rate (%)	Percentage of Fertilized Eggs (%)
0 (Control)	459 ± 18	457 ± 19	99.56 ± 5.32	100 ± 0.00
0.001	408 ± 13 *	387 ± 16 *	94.85 ± 4.38 *	96.81 ± 4.96 *
1	370 ± 15 **	312 ± 13 **	84.32 ± 2.15 **	88.92 ± 2.66 *
100	142 ± 8 **	45 ± 4 **	31.69 ± 0.86 **	45.77 ± 1.08 **

The experiments were repeated three times. The significance of differences is indicated by * *p* ≤ 0.05, ** *p* ≤ 0.01.
